# WO_3_ Photocatalyst Containing Copper Inactivates SARS-CoV-2 Pango Lineage A and Omicron BA.2 Variant in Visible Light and in Darkness

**DOI:** 10.3390/pathogens11080922

**Published:** 2022-08-16

**Authors:** Ryosuke Matsuura, Ken Maeda, Kyoji Hagiwara, Yosuke Mori, Toru Kitamura, Yasunobu Matsumoto, Yoko Aida

**Affiliations:** 1 Laboratory of Global Infectious Diseases Control Science, Graduate School of Agricultural and Life Sciences, The University of Tokyo, 1-1-1 Yayoi, Bunkyo-ku, Tokyo 113-8657, Japan; 2 Department of Veterinary Science, National Institute of Infectious Diseases, Toyama, Shinjuku-ku, Tokyo 162-8640, Japan; 3 Advintage Co., Ltd., 1-1-1-705 Ebisuminami, Shibuya-ku, Tokyo 150-0022, Japan; 4 Centre for Advanced Materials and Energy Sciences, Universiti Brunei Darussalam, Gadong BE1410, Brunei; 5 Laboratory of Global Animal Resource Science, Graduate School of Agricultural and Life Sciences, The University of Tokyo, 1-1-1 Yayoi, Bunkyo-ku, Tokyo 113-8657, Japan

**Keywords:** SARS-CoV-2 inactivation, Pango lineage A, Omicron variant BA.2, WO_3_ photocatalyst, time-dependency, dose-dependency, copper based disinfection, environmental disinfection

## Abstract

Severe acute respiratory syndrome coronavirus 2 (SARS-CoV-2) is the causative agent of coronavirus disease 2019, which has been a global pandemic. Since SARS-CoV-2 is transmitted through contaminated surfaces and aerosols, environmental disinfection is important to block the spread of the virus. Photocatalysts are attractive tools for virus inactivation and are widely used as air purifiers and coating materials. However, photocatalysts are inactive in the dark, and some of them need to be excited with light of a specific wavelength. Therefore, photocatalysts that can effectively inactivate SARS-CoV-2 in indoor environments are needed. Here, we show that a WO_3_ photocatalyst containing copper inactivated the SARS-CoV-2 WK-521 strain (Pango lineage A) upon irradiation with white light in a time- and concentration-dependent manner. Additionally, this photocatalyst also inactivated SARS-CoV-2 in dark conditions due to the antiviral effect of copper. Furthermore, this photocatalyst inactivated not only the WK-521 strain but also the Omicron variant BA.2. These results indicate that the WO_3_ photocatalyst containing copper can inactivate indoor SARS-CoV-2 regardless of the variant, in visible light or darkness, making it an effective tool for controlling the spread of SARS-CoV-2.

## 1. Introduction

Severe acute respiratory syndrome coronavirus 2 (SARS-CoV-2) is the causative agent of coronavirus disease 2019 (COVID-19), which has had an unprecedented impact on modern human civilization [1] and resulted in more than 6.3 million deaths globally as of early June 2022. Despite the development of drugs and vaccines, the number of infected people continues to increase. Although the transmission route of SARS-CoV-2 is still being debated, it is generally believed to be transmitted through the airborne route, surface contamination, and fecal-oral transmission [2]. Thus, the inactivation of the virus in the air and on surfaces is essential for controlling its transmission. In addition, the genome of SARS-CoV-2 has mutated rapidly, and several variants are reported. Mutations in the virus help it to evade the host immune system and to acquire drug resistance. Therefore, despite the presence of vaccines and drugs, it is important to find effective ways to inactivate the virus to prevent the spread of infection, regardless of the variant. It is reported that SARS-CoV-2 can be inactivated by photocatalysts [3], heat [4], ultraviolet (UV) light [5,6] and disinfectants such as ethanol [7]. In particular, since photocatalysts are harmless to the human body, unlike UV light, they have recently received great attention. It has been proposed that they can be applied for disinfection of living and working spaces without evacuating people.

Photocatalysts are excited by light and exhibit a strong oxidation-reduction reaction generating reactive oxygen species (ROS), such as hydroxyl (·OH) and superoxide radicals (O_2_^−^), on their surface [8]. Using this oxidation-reduction reaction, photocatalysts kill microorganisms, such as bacteria and fungi, and inactivate viruses such as influenza virus, hepatitis C virus, vesicular stomatitis virus, enterovirus, herpes virus, Zika virus, human coronavirus, bovine coronavirus, human norovirus, murine norovirus, SARS coronavirus, and bacteriophages [8,9,10,11,12,13,14,15]. Many compounds such as titanium dioxide (TiO_2_), tungsten trioxide (WO_3_), zinc oxide (ZnO), cadmium sulfide (CdS), and iron (III) oxide (Fe_2_O_3_) are known to exhibit photocatalysis and are being actively researched. In particular, TiO_2_ and WO_3_ have been reported to inactivate SARS-CoV-2, and are very promising as antiviral materials [3,10,16]. In addition, photocatalysts damage the viral morphology, RNA and proteins, leading to the inactivation of SARS-CoV-2 [3,16]. Therefore, it is expected that photocatalysts can inactivate SARS-CoV-2, regardless of the rapidly evolving variants.

On the other hand, photocatalysts have three limitations: First, the photocatalytic reaction occurs only on the surface of the photocatalyst. Therefore, it is necessary to coat all the surfaces to avoid contamination or to use it together with a circulator such as an air purifier. Second, the wavelength of light that can be used to excite the photocatalysts is limited. The wide bandgap (larger than 3 eV) of TiO_2_, which is the most common photocatalyst, limits the wavelength of the excitation light to the UV region [17]. Thus, narrowing the band gap of TiO_2_ is very important for using the TiO_2_ photocatalyst under visible light [18]. For example, mixing TiO_2_ with silicane (SiH) narrows the band gap (2.082 eV), and it can be excited with visible light [19]. Third, since light is necessary for the excitation of photocatalysts, the photocatalytic reaction does not occur in dark conditions, such as while sleeping. 

In this study, to overcome these limitations, we used a WO_3_ photocatalyst containing copper that can be applied to a surface by spraying. WO_3_ coating kept the surface clear of viral contamination. In addition, unlike TiO_2_, WO_3_ could be excited by room light even without mixing with any other compounds such as SiH. Therefore, a light source with a specific wavelength was not required. Furthermore, mixing copper with WO_3_ particles is expected to enable the photocatalyst to inactivate the virus even in the dark due to the effect of copper. However, there is only one study that reported the inactivation of the SARS-CoV-2 Pango lineage A by a WO_3_ photocatalyst [16]. In this study, we investigated the SARS-CoV-2 inactivation ability of a WO_3_ photocatalyst both in white light (irradiated by a light emitting diode (LED)) and in darkness, confirmed the time- and concentration-dependency of SARS-CoV-2 inactivation by the WO_3_ photocatalyst, and analyzed the effectiveness of this photocatalyst against different variants of SARS-CoV-2, according to Japanese Industrial Standards (JIS).

## 2. Results

### 2.1. Characterization of the WO_3_ Photocatalyst

**Figure 1:**
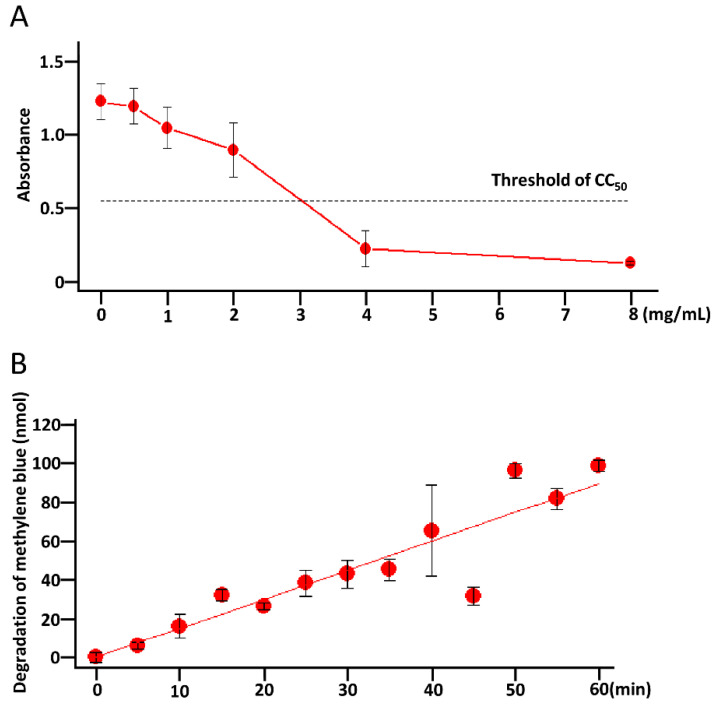
Cytotoxicity and methylene blue degradation potential of the WO_3_ photocatalyst. (**A**) The half-maximal cytotoxic concentration (CC_50_) was calculated from the water-soluble tetrazolium salt (WST-1) assay. Each dot and error bar represent the mean ± standard deviation (SD) from three independent experiments. (**B**) To confirm the ability of the WO_3_ photocatalyst to decompose organic matter, WO_3_-coated glass was placed in 25 mL of 12.5 μM of methylene blue and irradiated with white light. Methylene blue was collected every 5 min, and the absorbance at 660 nm was measured. The methylene blue contents were calculated from the OD value. The degradation speed of methylene blue was calculated from linear regression analysis.

First, to confirm the cytotoxicity of the WO_3_ photocatalyst, we added it to the culture media of Vero E6/TMPRSS2 cells and incubated the cells for 48 h. Cell viability was measured using the water-soluble tetrazolium salt (WST-8) assays. As shown in Figure 1A, the absorbance of WST-8 decreased in a concentration-dependent manner, which indicated that the WO_3_ photocatalyst affected cell viability modestly at the half-maximal cytotoxic concentration (CC_50_) of 3.0 mg/mL. In addition, the ability of the photocatalyst to decompose organic matter was confirmed in the methylene blue degradation assay. Methylene blue was degraded in a time-dependent manner at a degradation speed of 1.5 nmol/min. These results suggested that the WO_3_ photocatalyst was harmless and had a strong ability to decompose organic matter.

### 2.2. Inactivation of SARS-CoV-2 WK-521 Strain by the WO_3_ Photocatalyst

**Figure 2:**
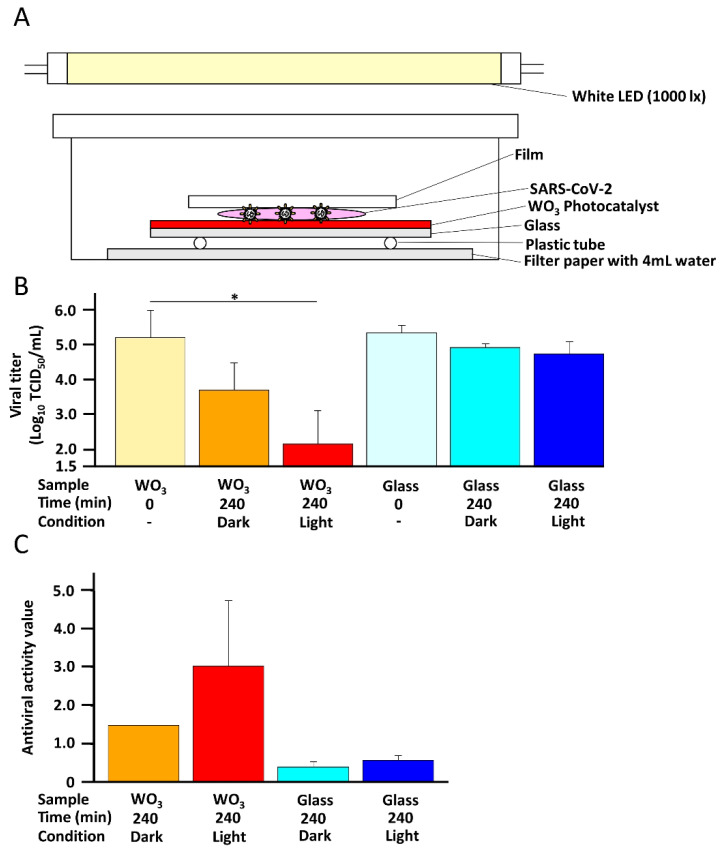
Inactivation of SARS-CoV-2 WK-521 strain by the WO_3_ photocatalyst. (**A**) A wet filter paper was placed in a 10 cm dish to avoid dryness. Glass with or without a coating of WO_3_ photocatalyst containing copper (100 mg) was placed on a plastic tube, which was in turn placed on the filter paper to avoid direct contact with the filter paper. SARS-CoV-2 WK-521 strain (150 μL) with a titer of 1 × 10^6^ 50% tissue culture infective dose (TCID_50_)/mL was placed on the coated or uncoated glass. The WO_3_ photocatalyst was excited by white LED light with 1000 lx for 0 or 240 min. To confirm the effect of WO_3_ photocatalyst containing copper in dark conditions, SARS-CoV-2 WK-521 strain was placed on the glass with or without a coating of the photocatalyst and kept in the dark for 240 min. (**B**) Titers of SARS-CoV-2 WK-521 strain were measured using the TCID_50_ assay with Vero E6/TMPRSS2 cells. Assays were performed in at least 6 wells, and the values represent the mean ± standard deviation (SD) of two independent experiments. Statistical comparisons were performed using Student’s *t*-test. Asterisk indicates a statistically significant difference (* *p* < 0.05). (**C**) Antiviral activity value was calculated using the formula: (the log_10_ titer of SARS-CoV-2 WK-521 strain of 240min sample) − (the log_10_ titer of SARS-CoV-2 WK-521 strain of 0 min sample of the same sample).

To confirm the inactivation ability of WO_3_ photocatalyst against SARS-CoV-2, the WK-521 strain was placed on the WO_3_ coated glass and irradiated with 1000 lx light (Figure 2A). As shown in Figure 2B, the titer of SARS-CoV-2 WK-521 strain placed on WO_3_ coated glass significantly decreased after irradiation with light for 240 min compared to before irradiation. In addition, the infectivity of SARS-CoV-2 WK-521 strain placed on WO_3_ coated glass in a dark place also decreased. However, this decrease was not to the extent observed in the illuminated sample. Indeed, the mean antiviral activity values were 3.04 and 1.50 with and without light conditions, respectively (Figure 2C). This decrement in the dark condition might be due to the antiviral effect of the copper contained in the WO_3_ photocatalyst. Indeed, the photocatalyst solution without WO_3_ exerted an antiviral effect on human coronavirus 229E (HCoV-229E) (Figure S1). On the other hand, no decrease in the titer was observed in the samples placed on the glass without WO_3_ coating for 240 min, with or without exposure to light. These results showed that the excitation light itself had no antiviral effect; the decrease in the titer was due to the effect of the LED-WO_3_ photocatalytic reaction in light and due to the antiviral effect of copper in darkness.

### 2.3. Time- and Dose-Dependency of the Antiviral Effects of WO_3_ Photocatalysts

**Figure 3:**
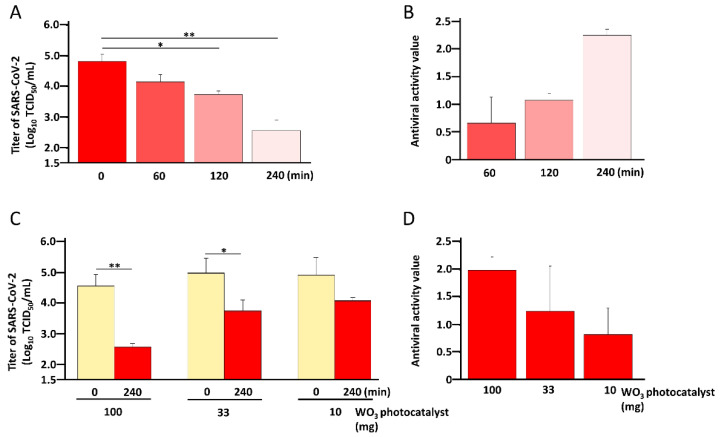
Time- and dose-dependency of the antiviral effects of WO_3_ photocatalysts. (**A**) To confirm time-dependency, SARS-CoV-2 WK-521 strain (150 μL) with a titer of 1 × 10^6^ 50% tissue culture infective dose (TCID_50_)/mL was placed on glass coated with WO_3_ photocatalyst (100 mg). WO_3_ photocatalyst was excited by white LED light with 1000 lx for 0, 60, 120 or 240 min. The titer of SARS-CoV-2 WK-521 strain was measured using the TCID_50_ assay with Vero E6/TMPRSS2 cells. Assays were performed in at least 6 wells, and the values represent the mean ± standard deviation (SD) of two independent experiments. (**B**) Antiviral activity value was calculated using the formula: (the log_10_ titer of SARS-CoV-2 of each time point sample) − (the log_10_ titer of SARS-CoV-2 of 0 min sample of same sample). (**C**) To confirm concentration-dependency, SARS-CoV-2 WK-521 strain (150 μL) with a titer of 1 × 10^6^ TCID_50_/mL was placed on glass coated with 10, 30 or 100 mg WO_3_ photocatalyst. WO_3_ photocatalyst was excited by white LED light with 1000 lx for 0 or 240 min. Titers of SARS-CoV-2 were measured using the TCID_50_ assay with Vero E6/TMPRSS2 cells. Assays were performed in at least 6 wells, and the values represent the mean ± SD of two independent experiments. (**D**) Antiviral activity value was calculated using the formula: (the log_10_ titer of SARS-CoV-2 WK-521 strain of 240 min sample) - (the log_10_ titer of SARS-CoV-2 WK-521 strain of 0 min sample of same concentration sample). Statistical comparisons were performed using Student’s *t*-test. Asterisk indicates a statistically significant difference (* *p* < 0.05; ** *p* < 0.01).

Next, to confirm the time-dependence of the antiviral effect of the WO_3_ photocatalyst, the SARS-CoV-2 WK-521 strain was placed on WO_3_-coated glass and irradiated with light for 0, 60, 120 and 240 min. As shown in Figure 3A, the viral titer decreased in a time-dependent manner, and the mean antiviral activity values were 0.66, 1.08 and 2.25 for 60-, 120- and 240-min light exposures, respectively (Figure 3B). In addition, to confirm the antiviral effect of the WO_3_ photocatalyst, the SARS-CoV-2 WK-521 strain was placed on glasses coated with 10, 30 or 100 mg of WO_3_ and irradiated with LED light for 0 or 240 min. As shown in Figure 3C, in the group irradiated with light for 240 min, a decrease in the titer was observed in all concentrations of WO_3_ photocatalyst compared to the group not exposed to light (0 min), which was significant in the 30 and 100 mg coatings. The antiviral activity values were 2.00, 1.25 and 0.83 for 100, 30 and 10 mg of WO_3_, respectively, indicating that the titer of SARS-CoV-2 WK-521 strain was decreased by the WO_3_ photocatalyst in a dose-dependent manner (Figure 3D). In contrast, there was no difference in viral titers among various concentrations on WO_3_-coated glass in the group which was not irradiated by LED light (0 min, Figure 3C). Our results demonstrated that photocatalysis is the mechanism of inactivation of SARS-CoV-2 WK-521 strain by WO_3_, photocatalytic inactivation of SARS-CoV-2 WK-521 strain by WO_3_ was dose- and time-dependent.

### 2.4. WO_3_ Photocatalysts Inactivate SARS-CoV-2 Omicron Variant BA.2

**Figure 4:**
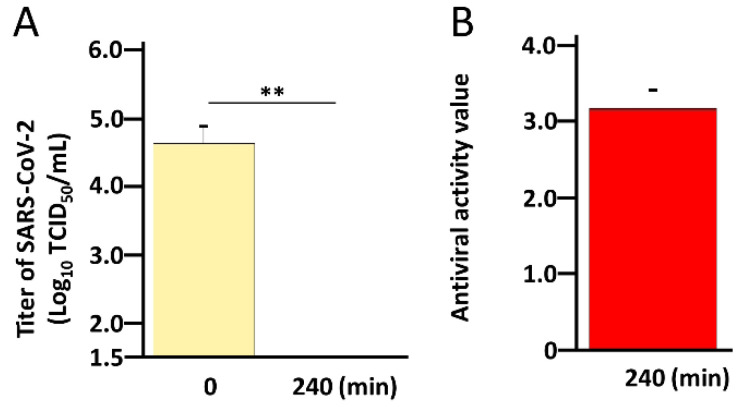
WO_3_ photocatalysts inactivate SARS-CoV-2 Omicron variant BA.2. (**A**) SARS-CoV-2 Omicron variant BA.2 (150 μL) with a titer of 1 × 10^7^ 50% tissue culture infective dose (TCID_50_)/mL was placed on glass coated with WO_3_ photocatalyst (100 mg). WO_3_ photocatalyst was excited using a white LED light with 1000 lx for 0 or 240 min. The titer of SARS-CoV-2 was measured using the TCID_50_ assay with Vero E6/TMPRSS2 cells. Assays were performed in at least 6 wells, and the values represent the mean ± standard deviation (SD) of two independent experiments. (**B**) Antiviral activity value was calculated using the formula: (the log_10_ titer of SARS-CoV-2 of 240 min sample) − (the log_10_ titer of SARS-CoV-2 of 0 min sample). Statistical comparisons were performed using Student’s *t*-test. Asterisk indicates a statistically significant difference (** *p* < 0.01).

Finally, we clarified whether the WO_3_ photocatalyst exerted an antiviral effect against SARS-CoV-2 Omicron variant BA.2. The Omicron variant BA.2 was placed on a WO_3_-coated glass (150 μL with a titer of 1 × 10^7^ 50% tissue culture infective dose (TCID_50_)/mL and irradiated with LED light for 0 to 240 min. As shown in Figure 4A, exposure to light for 240 min reduced the titer of this variant, similar to what was observed in the WK-521 strain. In addition, the mean antiviral activity after 240 min of photocatalytic reaction on WO_3_ was 3.17, which was comparable to that observed in the WK-521 strain (Figure 4B). This result indicated that the WO_3_ photocatalyst inactivates SARS-CoV-2 regardless of the variant.

## 3. Discussion

In this study, we demonstrated that the WO_3_ photocatalyst containing copper effectively inactivated SARS-CoV-2. Indeed, our results provide evidence that WO_3_ photocatalytic reaction for 240 min significantly decreased the infectivity of the SARS-CoV-2 WK-521 strain. Additionally, the copper present in the photocatalyst enabled it to inactivate the virus even in darkness. Furthermore, the WO_3_ photocatalyst containing copper decreased the SARS-CoV-2 WK-521 strain titers in a time-and dose-dependent manner, confirming the photocatalysis induced the inactivation of the virus. Our results are supported by a previous report that showed the effective inactivation of SARS-CoV-2 by a WO_3_-based visible light-responsive photocatalyst under different temperatures and exposure durations [16]. Notably, we demonstrated the effectiveness of a 240 min photocatalytic reaction involving the WO_3_ photocatalyst containing copper, not only against the SARS-CoV-2 WK-521 strain but also against the Omicron variant BA.2, as indicated by the decreased viral titers comparable with those of the WK-521 strain. These results suggest that the WO_3_ photocatalyst exerts an antiviral effect regardless of the variant. The present study is the first to report that a WO_3_ photocatalytic reaction can inactivate SARS-CoV-2, regardless of the variant.

It was previously reported that the mechanisms involved in the inactivation of SARS-CoV-2 by photocatalysis are damage to viral morphology, RNA, and protein [3,16]. In this study, the inactivation of the SARS-CoV-2 WK-521 strain and the Omicron variant BA. 2 by a WO_3_ photocatalyst containing copper was demonstrated. This suggests that even if the virus is mutated, a photocatalytic reaction by WO_3_ can achieve viral inactivation by damaging the viral protein, RNA and lipid bilayer, irrespective of the variant. Hence, this WO_3_ photocatalyst containing copper could be effective against potential variants of SARS-CoV-2 that may develop in the future. 

WO_3_ photocatalyst containing copper inactivated SARS-CoV-2 not only upon irradiation with light but also in dark conditions as well. It has been reported previously that copper nanoparticles can inactivate SARS-CoV-2 [20]. In addition, copper oxide nanoclusters grafted with titanium dioxide also inactivated SARS-CoV-2 alpha, beta, gamma and delta variants under illumination and in dark conditions as well [21]. These observations suggest that copper is responsible for the inactivation of the SARS-CoV-2 WK-521 strain under dark conditions observed in this study, indicating that the WO_3_ photocatalyst containing copper can be effective even at night time.

**Figure 5:**
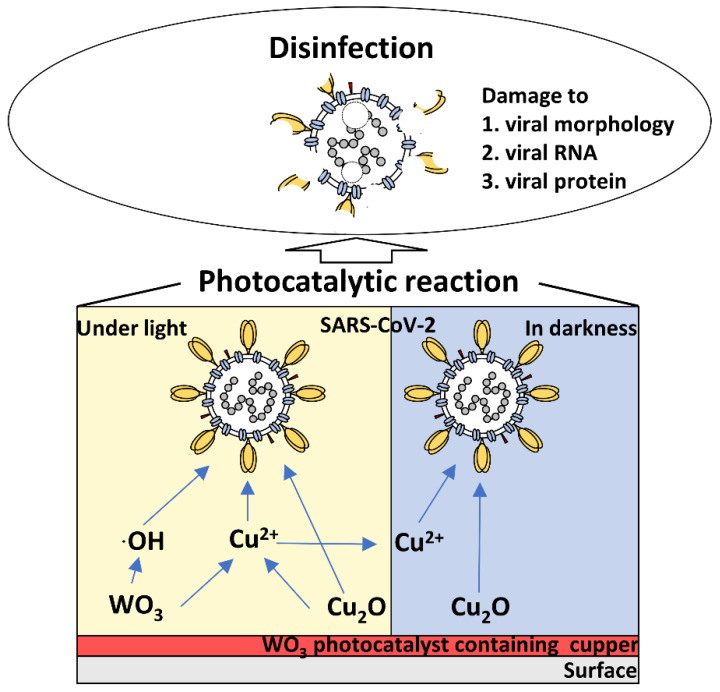
Schematic representation and the hypothesis of the inactivation mechanism of the WO_3_ photocatalyst containing copper. The findings of this study revealed that the WO_3_ photocatalyst containing copper inactivates SARS-CoV-2 under light or in darkness, regardless of the variant. Based on the results from previous studies, hydroxy radicals and copper ions considered to be generated by the WO_3_ photocatalyst and solid-state copper inactivate the virus under light, whereas in the dark, the virus is considered to be inactivated by the antiviral activity of solid-state copper and copper ions retained after the photocatalytic reaction [3,16,21]. In addition, the inactivation of SARS-CoV-2 might be attributed to the damage caused to the viral morphology, RNA, and proteins [3,16,21].

The WO_3_ photocatalyst containing copper can inactivate SARS-CoV-2 under light and in darkness, regardless of the variant. Findings from previous reports suggest the inactivation of the WO_3_ photocatalyst containing copper was owing to the damage caused to viral morphology, RNA, and proteins (Figure 5) [3,16,21]. Under light, this damage may be induced by hydroxy radicals and copper ions generated by the WO_3_ photocatalyst and solid-state copper. Conversely, in darkness, solid-state copper and copper ions retained after the photocatalytic reaction may inactivate the virus. The WO_3_ photocatalyst containing copper is considered to efficiently inactivate SARS-CoV-2 by these mechanisms, regardless of the variant.

Since a WO_3_ photocatalyst containing copper was excited by a white LED in this study, it is evident that this photocatalyst works effectively in an indoor environment without the necessity for any specific light source. In addition, unlike UV light, the WO_3_ photocatalyst is harmless to the human body. Although evidence from recent studies suggests that SARS-CoV-2 infection from contaminated surfaces is not as relevant [22,23,24], its possibility should not be ignored, especially when considering indoor spaces with a high probability of infection. Therefore, we may expect that the WO_3_ photocatalyst containing copper can be used for the disinfection of surfaces that are touched regularly by multiple individuals, such as the buttons on an elevator and straps of a train, to prevent viral spread. In addition, findings from our previous study showed that an air purifier with a TiO_2_ photocatalyst could inactivate SARS-CoV-2 in aerosols [3]. Therefore, the WO_3_ photocatalyst may also inactivate SARS-CoV-2 in aerosols if used in an air purifier. Thus, this study demonstrated that a WO_3_ photocatalyst containing copper could be effectively applied to control SARS-CoV-2 transmission and mitigate the ongoing COVID-19 pandemic.

## 4. Materials and Methods

### 4.1. Virus and Cell Culture

Vero E6 cells, which express the transmembrane serine protease TMPRSS2 (Vero E6/TMPRSS2 (JCRB1819), were maintained in Dulbecco’s modified Eagle’s medium (DMEM, Thermo Fisher Scientific, Waltham, MA, USA) supplemented with 10% heat-inactivated fetal bovine serum (FBS, Thermo Fisher Scientific) at 37 °C with 5% CO_2_. MRC-5 cells (CCL-171) were maintained in Eagle’s minimal essential medium (EMEM, Thermo Fisher Scientific) supplemented with 10% heat-inactivated FBS at 37 °C with 5% CO_2_. The WK-521 strain (Pango lineage A; 2019-nCoV/Japan/TY/WK-521/2020) [25] and Omicron variant BA.2 (hCoV-19/JPN/TY40-385/2022 strain) of SARS-CoV-2 were cultured and quantified using Vero E6/TMPRSS2 cells in DMEM containing 2% FBS. HCoV-229E was cultured and quantified in MRC-5 cells in DMEM containing 2% FBS. The infectivity of SARS-CoV-2 and HCoV-229E was calculated by their titration in Vero E6/TMPRSS2 cells and MRC-5 cells, respectively, using the TCID_50_ assay and the Reed–Muench method [26].

### 4.2. Cytotoxicity Assay

Vero cells (5 × 10^4^ cells/well) were seeded overnight in 24-well plates and treated with 0, 0.5, 1, 2, 4, or 8 mg/mL WO_3_ photocatalyst for 48 h. The cells were washed with PBS and cultured in 1 mL of fresh DMEM plus 30 µL of WST-8, and the WST-8 assays were performed at 37 °C for 90 min. The optical density at 450 nm (OD_450_) was measured using an Ensight Perkin Elmer multimode plate reader (Perkin Elmer, Milan, Italy). CC_50_ was calculated from the linear regression of OD_450_ vs. compound concentration.

### 4.3. Degradation of Methylene Blue

A glass coated with 100 mg of the WO_3_ photocatalyst containing copper (NFE2-W; Chemical Technology Co., Ltd., Takaishi, Japan) was put in 25 mL of 12.5 μM methylene blue solution. The WO_3_ photocatalyst was excited with a white LED (BBZ T13 Silver; Dongguan Oushi Electronic Technology Co., Ltd., Dongguan, China) every 5 min for 60 min. To confirm the effect of the WO_3_ photocatalyst on methylene blue, methylene blue was collected at each time point, and the absorbance at 660 nm was measured using an Ensight Perkin Elmer multimode plate reader. The degradation speed of methylene blue was calculated from the linear regression of OD_450_ vs. the photocatalytic reaction time.

### 4.4. Inactivation of SARS-CoV-2 and HCoV-229E by the WO_3_ Photocatalytic Reaction

The photocatalytic reaction was performed according to JIS R1752:2020 [27] with a minor modification (Figure 2A). Briefly, filter paper was placed at the bottom of the 10 cm dish and wetted with 4 mL sterilized water for moisture preservation. To avoid directly touching the filter paper, a plastic tube was placed on the filter paper, and glass coated with 100 mg WO_3_ photocatalyst containing copper was placed on top of the plastic tube. One hundred and fifty microliters of the WK-521 strain with a titer of 1 × 10^6^ TCID_50_/mL, Omicron variant BA.2 with a titer of 1 × 10^7^ TCID_50_/mL, or HCoV-229E with a titer of 1.47 × 10^5^ TCID_50_/mL was placed on the WO_3_-coated glass and spread by covering it with a film. Glass without the WO_3_ coating was used as a negative control. To confirm the antiviral effect of copper, glass coated with a photocatalyst without WO_3_ was used for HCoV-229E. The samples were then illuminated with 1000 lx light using a white LED for 240 min or not illuminated. After illumination, the samples were washed by immersing in 8 mL phosphate-buffered saline (PBS). As time control, the virus was immediately collected after placing the droplet on glass with or without WO_3_ coating (0 min). To confirm the time dependency, the SARS-CoV-2 WK-521 strain was placed on the 100 mg WO_3_-coated glass and illuminated for 0, 60, 120 and 240 min. To observe the concentration dependency, the SARS-CoV-2 WK-521 strain was placed on glass coated with 100, 30 or 10 mg WO_3_ and illuminated for 0 and 240 min. The SARS-CoV-2 and HCoV-229E titers in all experiments were measured using the TCID_50_ assay. 

The photocatalytic inactivation efficiency was defined as follows:

Antiviral activity value = log_10_ (*N_t_*) − log_10_ (*N*_0_) (1)

where *N_t_* represents the virus titer of the photocatalytically treated specimens after irradiation for t hours; *N*_0_ represents the virus titer of the photocatalytically treated specimens just after inoculation (0 min).

### 4.5. Statistical Analysis

Statistical comparisons were performed using Student’s *t*-test. *p*-values < 0.05 were considered statistically significant.

## 5. Conclusions

This is the first report showing that a WO_3_ photocatalyst inactivates Omicron variant BA.2 as well as the SARS-CoV-2 WK-521 strain, indicating the effectiveness of this photocatalyst against the virus, regardless of the variant. In addition, a WO_3_ photocatalyst containing copper can inactivate the virus using a simple white light or even in dark conditions, indicating its potential for wide application. In conclusion, a WO_3_ photocatalyst containing copper could be a very effective tool for controlling the spread of SARS-CoV-2.

## Data Availability

Not applicable.

## Supplementary Materials

The following supporting information can be downloaded at: https://www.mdpi.com/article/10.3390/pathogens11080922/s1, Figure S1: Antiviral effect of WO_3_ photocatalyst with or without WO_3_ for HCoV-229E.
