# Supplementary material for: WO3 Photocatalyst Containing Copper Inactivates SARS-CoV-2 Pango Lineage A and Omicron BA.2 Variant in Visible Light and in Darkness
Source: Pathogens. 2022 Aug 16;11(8):922. doi: 10.3390/pathogens11080922 (PMC9415178; doi:10.3390/pathogens11080922)
Supplement: Supplementary file 1 [file pathogens-11-00922-s001.zip › pathogens-1801945-supplementary.pdf]

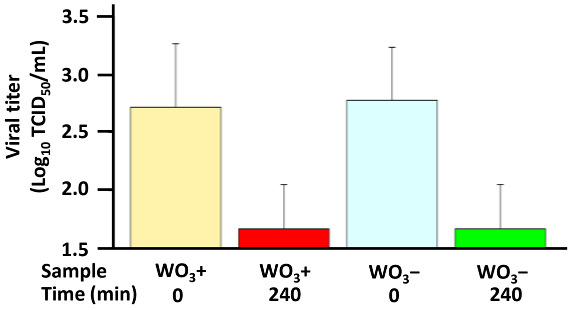

Figure S1: Antiviral effect of WO_3 photocatalyst with or without WO_3 for HCoV-229E. A wet filter paper was placed in a 10 cm dish to avoid dryness. Glass coated photocatalyst solution
with or with out WO_3 (100 mg) was placed on a plastic tube which was in turn placed on the filter paper to avoid direct contact with the filter paper. HCoV-229E (150 μL) with a titer of
1.47×10^5 50% tissue culture infective dose
($TCID_{50}$)/mL was placed on the coated or uncoated glass. WO_3 photocatalyst was excited by white LED light with 1000 lx for 0 or 240 min. Titers of HCoV-229E strain were measured
using the $TCID_{50}$ assay with MRC-5 cells. Assays were performed in at least 6 wells and the values represent the mean $\pm$ standard deviation (SD) of three independent experiments.
